# Impact of Perception of Green Space for Health Promotion on Willingness to Use Parks and Actual Use among Young Urban Residents

**DOI:** 10.3390/ijerph17155560

**Published:** 2020-08-01

**Authors:** Chongxian Chen, Weijing Luo, Haiwei Li, Danting Zhang, Ning Kang, Xiaohao Yang, Yu Xia

**Affiliations:** 1 College of Forestry and Landscape Architecture, South China Agricultural University, Guangzhou 510642, China; luoweijing@stu.scau.edu.cn (W.L.); hiwaylee@stu.scau.edu.cn (H.L.); 2 College of Landscape Architecture and Arts, Northwest A&F University, Xianyang 712100, China; zhangdanting@nwafu.edu.cn; 3 School of Architecture, Tsinghua University, Beijing 10084, China; ningkang@mail.tsinghua.edu.cn; 4 School for Environment and Sustainability, University of Michigan, Ann Arbor, MI 48104, USA; xiaohaoy@umich.edu

**Keywords:** green space, health promotion, park use, young urban residents

## Abstract

Promoting the use of green space is a fundamental way to improve physical and mental health and to enhance the quality of life of urban residents. In response to increasing demand for green space in cities, the impact of perception of green space for health promotion on willingness to use parks and actual use among young urban residents was investigated in this study. A total of 1135 young residents (ages 18–35) in three cities in China were surveyed by online questionnaire. A group of multiple regression models was constructed to investigate the influencing perception factors of participants’ willingness to use parks and actual use. The results revealed that the young residents’ perception of green space components for health promotion (green space access, types, sizes, plants, water, sensory features, microclimate environments and amenity facilities) had a greater effect on their willingness to use parks and to promote health, while it was less influential with respect to their actual park use behavior (frequency and duration). Among these variables, green space access is a critical concern for willingness to use toward parks. The disparities of perception of green space for health promotion effect on willingness to use a park and actual use provide a better understanding of the psychological factors affecting park use among young residents. The findings also provided some implications for public health policymakers, urban planners and landscape architects in designing parks to encourage visitation by young people.

## 1. Introduction

As urbanization and populations rapidly increase, maintaining livable and healthy cities has become a great challenge around the world [1]. The inactive urban lifestyle is thought to be associated with increases in hypertension [2], diabetes [3], obesity [4] and mental health issues such as depression and anxiety [5]. It has been suggested that today’s younger generations are less healthy and more likely to suffer from chronic diseases than their predecessors [6]. In China, urban environments have led to changes in lifestyles that increase the risk factors for noncommunicable diseases [7]. To address urban-related health problems, various preventative measures have been proposed in urban environments [8,9,10]. Providing urban green spaces has become one of the important measures to promote physical activity and enhance human well-being, which has aroused wide interest among researchers and policymakers [11,12,13].

Green spaces such as urban parks are a useful environmental source for urban dwellers to improve physical, mental and social health [14]. For example, interactions with urban parks can encourage physical activity, thereby reducing heart disease, type II diabetes and obesity and thus improving the physical health of people [15,16]. Natural landscapes in urban parks reduce mental fatigue and help people find relief from their stressful daily routines, as well as improve cognitive capacity [17,18,19]. Furthermore, urban parks provide a variety of opportunities for people to participate in public activities and thus promote social interaction [20,21].

Despite the health benefits associated with urban park visitation, recent studies have shown that parks are generally underutilized among young people [22,23]. To date, most of the empirical studies on the associations between park usage and health outcomes for young people have been focused on Western countries in urban contexts [24,25,26]. However, it is difficult to compare these relationships observed in other countries to the Chinese population. First, the differences in park physical features between cities in China and other countries could result in different relationships with park usage among residents [27]. Second, a large amount of evidence shows that people from different ethnic and cultural backgrounds and ages vary in the ways they use green spaces and in their perceptions of the beneficial effects of parks [28,29,30,31]. Consequently, it is necessary to understand these relationships in a Chinese context.

Although the literature concerning the factors affecting park usage is accumulating in China, most studies focus on the objective environment attributes, such as park pathways [32,33], quality of greenery [34] and accessibility [35,36]. Studies examining the psychological mechanism of built environments on individual’s park use behavior are still insufficient. A few studies suggest that peoples’ perception of the environment could influence willingness and intention toward their surroundings, which may stimulate actual behavior [37,38]. Relatively fewer studies pay attention to the effects of perception of green space for health promotion on resident willingness to use parks. Furthermore, as cities usually with higher living standards, many large cities in China have attracted large population of young residents that are facing more pressures than their predecessors (e.g., education and employment), and the health status of this group has become a great social challenge [39,40]. However, existing studies related to the association between psychological factors and park usage for young urban residents remain relatively deficient in Chinese society. This is important because encouraging park visitation could promote the physical health and mental health of young residents [41].

Aiming to address the above knowledge gaps, the associations among young urban residents’ perception of green space for health promotion, willingness to use parks and actual behavior in three cities in China were investigated. Young urban residents age ranging from 18 to 35 years were selected according to the definition of young residents from previous research and China’s medium and long term youth development plan (2016–2025) [34,42,43]. In addition, owing to the recent changes in the role of traditional parks, new and more accessible forms of green space have been gaining more attention among urban residents. Therefore, green spaces such as roof gardens, green lane and greenery associated with housing estates were added into the broader context of parks in our study. Based on the study results, several suggestions for Future Park planning and design to encourage visitation by urban young residents are proposed herein.

## 2. Literature Review and Theoretical Framework

### 2.1. Perceived Benefit and Willingness to Use toward Green Space

Perceived benefit relates to an individual’s perception of the positive changes that are brought by specific behavior, which, in turn, influences the attitude of a person toward their external environment [38,44]. Additionally, according to the theory of planned behavior (TPB), willingness is affected by attitude, i.e., if a person’s attitude toward a certain behavior is more positive, the person then has a stronger willingness to exhibit that behavior [37].

Many studies have demonstrated the roles of perception in contributing to behavioral intention towards green space [45,46,47]. For example, Chen et al. showed that the perception of benefits of green space is closely associated with the willingness of residents to conserve green space [48]. Perceptions of the contribution of small green spaces in reducing air pollution had a positive impact on residents’ willingness to pay for maintaining roof gardens and green lanes [49]. Furthermore, some studies have noted that awareness of the benefits that green space provide may increase the willingness of the people to use green space [46,50]. The presence of water features in a park could afford relaxed feelings and reduces stress; hence, people would have a positive attitude toward the park, ultimately enhancing their willingness to visit parks [51,52]. Additional studies in Western countries also suggest that the perceptions of greenness density, biodiversity, presence of old trees, comfort, cleanliness and order could indirectly influence an individual’s intention to use parks [53,54]. For example, perceived amount of vegetation could promote use of space and contribute to the fostering of social activities [55]. Perception of park cleanliness and order have also been found to be strongly related with frequency of visitation [56]. However, a negative perception of the green space may contribute to a negative willingness to use it. The perception of fear or insecurity in a park could provoke a psychological barrier that makes people avoid the park [57]. To some extent, such an insecure feeling has an adverse impact on attitudes toward the willingness to use parks [58]. In addition, evidence indicates that differences exist between Chinese and Western attitudes towards urban parks and green spaces, e.g., park usage pattern and outdoor recreation characteristics [30]. Therefore, it is likely that differences between Western and Chinese young urban residents influence the perception of benefits of park use, which in turn vary in promoting their willingness towards park use for improving health.

### 2.2. Factors Affecting Park Use

During the past several decades, many studies have been conducted by scholars to explore factors that affect the interactions between people and urban green space. Structural factors, such as size and proximity, have demonstrated associations with park use [59,60]. For example, Cohen et al. found that increase in parkland size was associated with an increase in the frequency of park visitation through a study of 174 neighborhood parks across the United States [61]. Other studies suggested that accessibility is an essential precondition for park use [62,63], as increased in distance from a park likely leads to a decline in the park visitation [64]. Park physical features may also play a vital role in encouraging park use or physical activity among residents [65], especially for specific features, e.g., playgrounds [66], water features [67] and vegetation [68]. In contrast, other features, such as litter, vandalism and poor playground surface quality have been shown to have a negative impact on park use [69,70].

In addition to physical factors, numerous studies have demonstrated the effects of psychological factors on the park use. For example, Ries et al. found that higher-quality perception of a park had a positive influence on park visitation among young people [71]. Some studies also showed that an individual’s nature orientation or attitude towards nature was the more important factor explaining urban dwellers’ actual park visitation [72,73]. Perceived vegetation quality and provision of facilities also encourage park visitation of residents, which in turn may improve mental, physical and social health [74,75]. Moreover, it was suggested that perceived accessibility was more important than a physical distance in determining park use behavior [76]. In view of the above, the roles of both physical and psychological factors in influencing park visitation are relatively well investigated, yet very little is known about the associations between young residents’ perception of green space for health promotion and their actual park use behavior.

**Figure 1:**
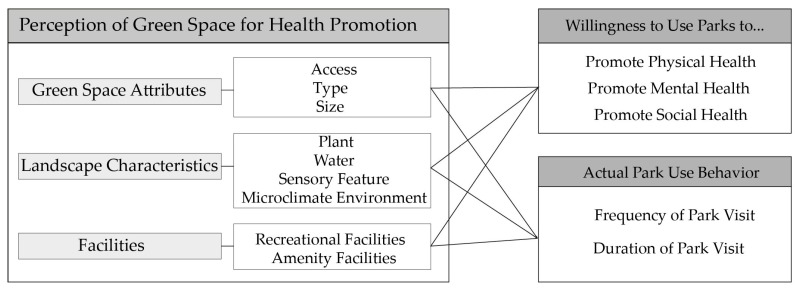
Potential relationships among perception of green space for health promotion, willingness to use parks and actual park use behavior among young urban residents.

Based on the literature review above, the theoretical framework in Figure 1 is proposed. Figure depicts how the perception of green space for health promotion affects willingness to use and actual use toward parks among urban young residents, in order to answer the following questions:

(1) Did the young residents’ perceptions of green space for health promotion shape their willingness to use park to promote health?

(2) Did this perception affect their park use behavior?

(3) Which factors of the perception of green space for health promotion were strongly associated with park use willingness and actual behavior?

The answers to these questions are important because they could increase our understanding of factors that contribute to the promotion of park use. Thus, urban policymakers could understand how to design and manage urban parks, which would effectively increase residents’ willingness to use parks, promote actual park use behavior and improve the health of young urban residents.

## 3. Materials and Methods

### 3.1. Study Design and Participants

This study was designed to improve understanding of the effect of young urban residents’ perceptions of green space components for health promotion on their park use willingness and behavior in Chinese cities. To achieve the objectives, an online questionnaire survey was conducted in three typical cities, i.e., Beijing, Xi’an and Guangzhou.

**Figure 2:**
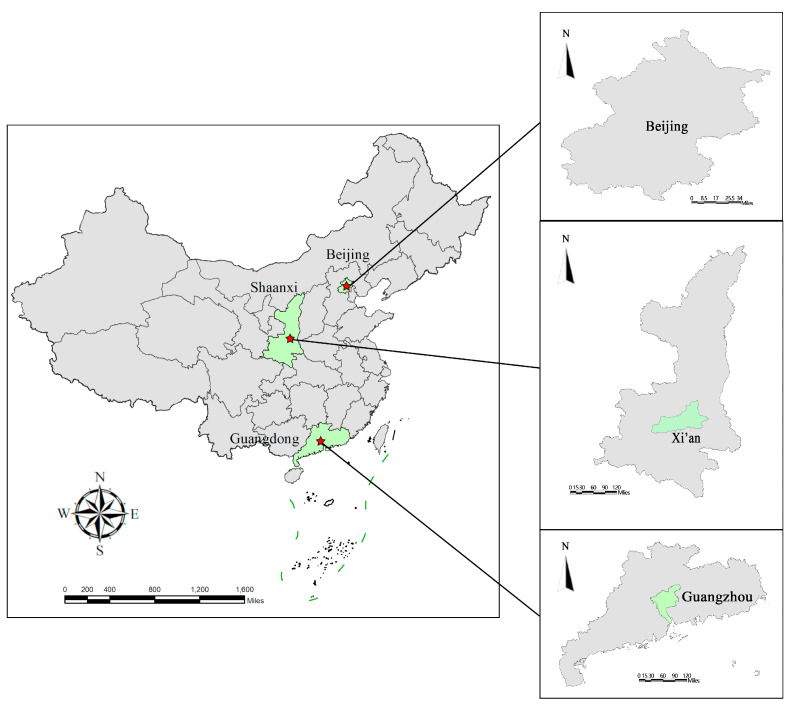
Locations selected for conducting the questionnaire.

Beijing, Xi’an and Guangzhou are in the Beijing–Tianjin–Hebei region, the Guanzhong Plain region and Pearl River Delta, respectively (Figure 2). The three cities offer important sites with which to investigate the impact of perception of green space for health promotion on willingness to use parks and actual use among young urban residents because of their demographics and green space distribution. Beijing, the capital of China, is the country’s political, cultural and economic center of China. By the end of 2018, Beijing had 21.53 million permanent residents [77]. The city occupied a total area of 16,410 km^2^ and had 852.86 km^2^ of green space and its official ratio of park green space was 16.3 m^2^ per capita [78]. The overall number and area of urban parks in Beijing has increased year-by-year. By 2015, the number of registered parks in Beijing has grown to more than 400, including Chaoyang district, Haidian district and Fengtai districts, which cover a large area [79]. Located in a river valley, Xi’an lies north of the Weihe River and south of the Qinling Mountains. Xi’an is an important megalopolis and a crucial scientific, educational and industrial base of Central China. As the capital of Shaanxi Province, Xi’an includes an area of 10,097 km^2^ and has a population of approximately 10 million. The urban green coverage of Xi’an reached 41.6% with 104 urban parks and a per capita park green space of 11.9 m^2^ by the end of 2018 [80]. Guangzhou is an important central city, international business center and comprehensive transportation hub of South China. As the capital of Guangdong Province, Guangzhou is a megacity with a population of nearly 15 million in 2018. It has a large percentage of urban green spaces with approximately 400 parks in the inner and outer suburbs, and the park green space per capita has reached 17.3 m^2^ [81]. With similar green coverage rates, diverse types of green spaces, populations and economic statuses, Beijing, Xi’an and Guangzhou are typical representatives of socioeconomic centers with the conditions to attract and retain youth in China, representing North, Central and South China [82]. Therefore, the sample data from these areas may mirror the willingness to use parks to promote health, park use behaviors and the components concerning perceptions of green space among young urban residents in China.

By March 2020, China had 904 million Internet users, among which about 80% were young people aged from 10 to 49 years old [83]. Therefore, an online questionnaire survey was a better choice for young urban residents that were more likely to have good access to the Internet than other age groups. The questionnaires on the Wenjuanxing website (www.wjx.cn), one of the most frequently used online data-collection platforms in China. Invitations were sent to potential subjects and responses solicited through social media and email from October 2017 to November 2019. Participants were informed that the eligible age for this study was between 18 years old and 35 years old [34,42,43], and participants must have lived in that city for at least six months. Respondents were reassured in advance that all identifying information would be kept anonymous and that the information was for noncommercial use. Finally, a total of 1340 online questionnaires were collected. The returned questionnaires had the quality criteria of all the questions being completed and verifiable demographic information being provided. Of all the collected questionnaires, 205 were invalid due to incompletion or being ineligible. Thus, the valid sample consisted of 1135 respondents, with an overall valid-response rate of 84.7% (the invalid-response rate was 15.3%).

### 3.2. Survey Instrument

A web-based questionnaire containing 20 questions in total was designed for data collection following an extensive literature review. It was divided into three subsections: (1) respondents’ willingness to use parks to promote health and actual park use behavior, (2) perception of green space components for health promotion and (3) demographic information. The first section was designed to capture the data for the dependent variables, including the willingness to use parks to promote health (three questions) and park use behavior (two questions). The second section included questions designed to gather participants’ perceptions about green space components for health promotion (nine questions). The third section was set to collect demographic information (six questions), including the city of residence, age, sex, education, monthly income and type of residence. It took approximately 5 to 10 min for each respondent to complete the questionnaire.

#### 3.2.1. Dependent Variables

According to the World Health Organization, “health is a state of complete physical, mental, and social well-being and not merely the absence of disease or infirmity [84].” Thus, the respondents’ willingness to use parks to promote health consisted of three categories, i.e., physical, mental and social health. These categories represent different dimensions of individual health that people would be willing to promote by going to green spaces. Physical health described the ability to protect and restore an organism (e.g., pressure and heart rate) from the changing circumstances of an external environment [85,86,87]. mental health was defined as the capacity to deal with, recover from and prevent psychological stress (e.g., anxiety and depression), as well as recognize one’s own intellectual and emotional potential [86,87,88]. Social health could be operationalized in terms of social support, referring to the ability to harbor good interpersonal relationships and social adaptation (e.g., interaction and collaboration) [85,86,87]. The study was used to investigate respondents’ willingness of park use to promote health by asking “Would you like to go to parks to improve your physical/mental/social health?” A five-point scale (from 1 = not at all to 5 = very willing) was implemented for the analysis to create the willingness index for health benefits.

Frequency and duration of the park visits were used to measure park use behavior. Frequency and the duration of park visits have been widely applied in studies of the relationship between green space and health [59,89,90]. The frequency of park visits was assessed by asking, “How often do you visit urban parks or other green spaces?” The response options for the first question were on a seven-point scale (1 = annually or less often, 2 = quarterly, 3 = monthly, 4 = biweekly, 5 = weekly, 6 = semiweekly and 7 = daily). The duration of park visits was measured by asking, “How long on average do you stay in your most frequently used parks?” The response options for the second question were on a five-point scale (1 = 30 min or less, 2 = 0.5–1 h, 3 = 1–2 h, 4 = 2–3 h, 5 = over 3 h).

#### 3.2.2. Independent Variables

The perceptions of green space components for health promotion included the perception of green space attributes (three items), landscape characteristics (four items) and facilities (two items). Three categories of green space components were adopted from previous studies [34,89,91,92,93,94,95]. For the green space attributes, participants were asked whether green space access (e.g., walking distance and road system), type (e.g., green space in residential areas and multifunctional parks) or size (e.g., 1, 2 and 5 Ha) played a role in respondents’ health promotion. The landscape characteristics were measured by asking whether plants (e.g., ornamental features and canopy density), water (e.g., artificial and natural), sensory features (e.g., sound and smell) or microclimate environments (e.g., temperature and humidity) contributed to health promotion. The facilities were assessed by asking respondents whether rest facilities (e.g., seats and pergolas with chairs) or amenity facilities (e.g., exercise facilities, running paths, swings and slides) had an impact on their health promotion. All the response options were listed on a Likert scale (1 = strongly disagree to 5 = strongly agree).

### 3.3. Statistical Analyses

Amos 21 software (IBM, Armonk, NY, USA) and SPSS (version 22.0, IBM, Armonk, NY, USA) was used for the statistical analysis in this study. First, the Cronbach’s α coefficients were examined and a confirmatory factor analysis (CFA) performed for the reliability and convergent validity of the constructs. Composite reliability (CR) coefficients and average variance extracted (AVE) scores were calculated as estimates. Second, the descriptive statistics were examined for both the demographic characteristics and the experimental variables. Third, Spearman’s correlation analysis was conducted to investigate the univariate correlations between the dependent variables (perceptions of green space components for health promotion and demographic variables) and independent variables (willingness to use parks and actual park use behavior), as well as screen irrelevant variables (*p* > 0.05). Finally, to further understand the influencing perception factors of young residents’ willingness to use parks for health promotion and their park use behavior, a set of multiple regression models was built. Additionally, collinearity statistics were applied to check multicollinearity issues if there were moderate to strong correlations between the independent variables. All independent variables fell within an acceptable level of the variance inflation factor (VIF) ratio (<4.0), implying that the concerns regarding multicollinearity were eliminated [96].

## 4. Results

### 4.1. Reliability and Validity Analysis

Regarding the reliability, the Cronbach’s α values for the willingness to use parks to promote health and the park use behavior were 0.836 and 0.712, respectively. As shown in Table 1, the Cronbach’s α values of the independent variables were all above the threshold of 0.7, which can be regarded as reliable. Regarding the convergent validity, the CR coefficients for each independent variable exceeded the threshold value of 0.7 [97] and average variance extracted (AVE) scores were calculated with values higher than 0.5 in Table 1 [98], indicating that the results were acceptable. Loading values, also called standardized path coefficients, were used to assess the relationship between variables, ranging from 0.677 to 0.912 among the independent variables in this study.

### 4.2. Descriptive Statistics

Table 2 displays the demographic and socioeconomic characteristics of the 1135 residents that participated in the online survey. More than half of the participants were young residents between the ages of 18 and 25 (60.5%) and most of the respondents were female (67.9%), which may be due to the fact that women are more likely to have a greater willingness to participate in online surveys than men [99]. Most of the participants had earned a bachelor’s degree (66.3%) or a master’s degree (24.8%), representing a relatively high educational level. In addition, 33.6% of respondents had a monthly personal income of less than 1000 CNY, followed by 19.7% with a monthly income of 5000 CNY to 10,000 CNY. Regarding the residence location, 61.8% of the participants reported that they were living in the central city, while 34.4% were living in suburban districts.

Table 3 lists the descriptive statistics for the dependent and independent variables used in the multiple regression models, including means and standard deviations. In terms of the willingness to use parks to promote health, the average scores for the physical health, mental health and social health variables were 4.04 ± 0.97, 4.42 ± 0.87 and 4.00 ± 0.99, respectively, ranging from willing to very willing, indicating that most of the participants were willing to go to parks to promote physical, mental and social health. Notably, among these three variables, the mental health variable had the highest mean scores.

For park use behavior, the mean score of the frequency of the park visits was 3.42 ± 1.37, ranging from monthly to biweekly, and the duration of the park visits was the lowest, between 0.5 h and 2 h (M = 2.23 and SD = 0.82).

For the perception of green space components for health promotion, the mean scores of all of the variables were higher than 4 on a five-point scale (M = 4.07–4.28), except for the rest facilities (M = 3.84 and SD = 1.07). This result indicated that most of the young residents reported relatively high awareness levels of the role of green space components for health promotion. However, the assessment of the role of rest facilities for health promotion ranged from neutral to agreement.

### 4.3. Correlation Analyses

Table 4 presents the bivariate correlation coefficients of the variables in this study. All of the perception variables (i.e., perception of green space components for health promotion) were positively and significantly correlated with the willingness to use parks to promote health (rs = 0.307–0.538 and ps < 0.01). Similarly, all the perception variables were positively associated with the frequency of park visits (rs = 0.109–0.161 and ps < 0.01). Moreover, almost all the perception variables were significantly associated with the duration of the park visits (rs = 0.060–0.115 and ps < 0.01), except for the green space access variable (r = 0.045 and *p* > 0.05). Some demographic variables (age, education level and income) had a significant correlation with the dependent variables (the willingness to use parks to promote health and the duration of the park visits). After filtering the variables, the perception variables significantly correlated with the dependent variables were included in the regression models. Some demographic variables (age, gender, education level and income) were also included in the regression model, since they may impact on the respondents’ park use willingness and behavior. In addition, there were moderate to strong correlations between the independent variables. Thus, the collinearity problem merited further investigation.

### 4.4. Predictors of the Willingness to Use Parks to Promote Health

Table 5 presents the multiple regression models using the willingness to use parks to promote health (i.e., physical, mental and social health) as the dependent variable. The results demonstrated that all of the variance inflation factor (VIF) values were less than 3.4, suggesting that the multicollinearity problem among the independent variables were acceptable.

With respect to the physical health variable, Model 1 explained approximately 45.7% of the variables. Green space access (β = 0.289, *p* < 0.01), type (β = 0.109, *p* < 0.01), size (β = 0.179, *p* < 0.01), sensory features (β = 0.129, *p* < 0.01) and amenity facilities (β = 0.079, *p* < 0.05) exerted a significant positive influence on the willingness to use parks to promote physical health. For the mental health variable, Model 2 accounted for approximately 57.3% of the variables. The results revealed that green space access (β = 0.301, *p* < 0.01), type (β = 0.098, *p* < 0.01), size (β = 0.172, *p* < 0.01), plants (β = 0.130, *p* < 0.01), sensory features (β = 0.136, *p* < 0.01) and microclimate environments (β = 0.107, *p* < 0.01) were positive predictors of the willingness to use parks to promote mental health. For the social health variable, Model 3 explained nearly 41.9% of the variables. Green space access (β = 0.229, *p* < 0.01), type (β = 0.151, *p* < 0.01), size (β = 0.151, *p* < 0.01) and water (β = 0.174, *p* < 0.01) demonstrated positive relationships with the willingness to use parks to promote social health and gender produce significant observations (β = −0.064, *p* < 0.05). In total, these predictors for Models 1–3 had significant explanation power, and all of the F-statistics were at the 0.01 level. In Models 1–3, the perception of green space access for health promotion was found to be the strongest predictor of willingness to use parks to promote health. The role of several demographic variables in the willingness to use parks to promote health was notable. Young men tended to report higher willingness to use parks to promote physical health (β = −0.058, *p* < 0.05) and social health (β = −0.064, *p* < 0.05), which was consistent with a previous study [100]. People who earned less monthly personal income were more willing to use parks to promote mental health (β = −0.061, *p* < 0.05), which may be explained by the fact that people with higher income could have multiple choices with which to promote health that were not restricted to park usage [101].

### 4.5. Predictors of Park Use Behavior

As shown in Table 6, we constructed regression analyses to examine the predictors of park use behavior. The results in Model 4 indicated that age (β = 0.099, *p* < 0.05), gender (β = −0.067, *p* < 0.05) and green space access (β = 0.097, *p* < 0.05) showed a significant influence on the frequency of park visits. In Model 5, plant (β = −0.102, *p* > 0.05) and microclimate environments (β = 0.120, *p* > 0.05) appeared statistically significant. However, the R-square values for Models 4 and Model 5 were less than 0.04, suggesting that the relationships between the predictors and the dependent variables were extremely weak, which also could be seen as having no relation. Thus, the predictors of the frequency and duration of park visits could not be determined from the perception of green space components for health promotion among young residents.

## 5. Discussion

### 5.1. Effects of Perception on Park Use Willingness and Behavior

The research reported in this article was intended to examine the impact of perception of green space components for health promotion on willingness to use parks and actual use among young residents in Chinese cities. Overall, the results revealed that young residents’ perception of green space components for health promotion had a greater effect on their willingness to use parks to promote health. In contrast, young peoples’ perceptions of green space components for health promotion were less influential on their actual park use behavior. The findings in this study provided a greater understanding of how the perception of parks for health promotion affected park use among young urban residents.

The park use willingness models that were examined in this study were moderately strong and the young residents’ perceptive assessments of eight specific green space components for health promotion were found to be positive predictors, including green space access, type, size, plants, water, sensory features, microclimate environments and amenity facilities. Notably, the perception of green space attributes for health promotion, including the green space access, type and size, had a positive effect on the intention to use parks for all three dimensions of health effects. This finding indicated that young residents that knew these three attributes of green space were crucial factors that would contribute to well-being were more willing to use parks to achieve physical, mental and social health. Moreover, the perception of “green space access” was the most robust predictor for park use willingness in this research. This may have been largely attributed to the reason that having access to green space was a prerequisite for health promotion realization, making it a more remarkable predictor [94]. The above findings were similar to those of previous studies, indicating that park utilization and future visitation intention related to health was positively determined by perceived accessibility [46,102,103]. Likewise, some research studies found that the type and size of green space were correlated with different park benefits, activities and park use [46,60,92,104,105]. For example, Byrne et al. reported that an individual’s perception of park accessibility was a determinant of park use decisions [102]. Brown et al. suggested that neighborhood parks were associated with psychological benefits, while community parks and natural parks were related to social benefits and environmental benefits, respectively [104]. Moreover, large green spaces tend to have more natural elements, recreational facilities and walking paths [106,107,108], which may offer more attractiveness and opportunities for various activities. Therefore, the health values and intentions that residents held for park use may have been related to these properties, which may partly explain the results in this study.

With respect to the perception of landscape characteristics for health promotion, there were three interesting findings. First, both the “plants” and “microclimate environments” variables were positive predictors of the willingness to use parks to promote mental health, suggesting that young residents could be more willing to visit parks to achieve psychological recovery if they believed that plants and microclimate environments played an important role in promoting health. The effect of plants on mental health has been widely recognized, and it has also been found to be a driving and attractive factor for the motivation to visit parks [72,109,110,111]. It has also been reported that planting influences microclimate environments and may lead to great well-being, relaxation and comfort [112,113], which possibly provokes higher intentions to visit parks. Second, the perception of water was associated with the willingness to use parks for social health. This result provided support for the established evidence that one of the main benefits people identified receiving from blue space visits was social interaction [114]. Additionally, the residents who had a better understanding of the importance of sensory features for health promotion reported a higher level of willingness to use parks for physical and mental health. Our research on the perception–willingness models may further expand the previous findings, which indicated that sensory perception was linked to health-related behaviors and mental restoration [115].

With respect to the perception of facilities for health promotion, the “amenity facilities” variable showed a significant impact on the willingness to use parks to promote physical health. The important role of amenity facilities in park use has been demonstrated in previous research, which may also explain the relationship found in this study [89,116].

However, the park use behavior models had weak and mixed results. It was found that the young residents’ perception of green space components for health promotion could not predict the frequency and duration of park visits. The results of this research also showed that most of the young residents reported relatively high awareness of the role of green space components for health promotion. They also showed high intensions to visit parks to promote health, especially mental health. However, most respondents tended to visit parks at low frequency, and they spent less of their time visiting parks. This was consistent with previous research studies that showed that parks were generally underutilized by young people [23,117].

The more noteworthy findings of this research were the disparities between young residents’ park use willingness and their use behavior. The results may have been partly explained by the following reasons. It was previously reported that young Chinese residents aged 20–30 were less likely to visit parks for leisure in their daily life because they preferred to pursue more active and exciting activities, such as visiting pubs and exploring wild areas [118]. Some potential barriers may influence young people’s actual park use behavior, such as less leisure time due to being busy with work and study [119,120]. Furthermore, insufficient availability of green space due to governmental and social failures or accessible barriers (such as entrance restrictions) may have an impact on the residents’ willingness and usage of green spaces [121]. In addition, according to previous research, young people spend most of their leisure on the Internet, rather than outdoor activities [122]. Moreover, the planning and design of urban parks in China have mainly focused on the recreational, esthetic and environmental effects, which may lack attractiveness for young residents.

However, our findings were different with some studies in the Western countries. First, unlike Chinese young adults, residents in western countries tend to visit green spaces more frequently. For example, a nationwide study in Denmark suggested that 43.0% of the adult Danes visit green space every day and 91.5% of them visit green space at least once per week [123]. This could be partly explained by the fact that many green spaces in Europe and America contained more physically challenging facilities that could encourage young people to visit green spaces, such as skateparks, climbing frames, running tracks, fitness rings,, etc. [24]. Conversely, there were fewer sports theme parks that could provide appropriate activities for young people in China [124]. Second, western people prefer to walk dogs, do ball sports, jog or enjoy the sunshine in green spaces [125,126]. However, young Chinese people seldom go to green spaces for activities, and they may think that green spaces are somewhere occupied by older people and children. Finally, compared to Western countries, the development of urban parks in China got off a late start. The residents of most Western countries have paid more attention to community parks and informal green spaces in recent years [127,128], while the priority of the planning and management were still large urban parks in Chinese green space departments, which may limit the usage of green space among young people.

### 5.2. Implications for Practice

This study generated knowledge that young residents’ perceptions were an important factor significantly affecting the willingness to use parks to promote health. The findings in our research fit into a broader picture of effects to facilitate park use for health promotion and the findings provide some implications for green space design. The significant perceptive predictors of the willingness to use parks to promote health could be incorporated more appropriately into the design of green space. For example, access to green space was found to be a crucial factor affecting park use. Thus, it is recommended that urban planners improve the physical and perceived accessibility of green spaces. Intervention strategies were suggested, such as making green spaces of different sizes and functions that would be reasonably distributed in the city scales and providing information about different routes to access parks for young residents [129]. Green space designers and managers also must pay more attention to other predictors, including plants, water, sensory features, microclimate environments and amenity facilities.

The findings in our study suggest that improving the above features may lead to higher willingness to use parks among young residents. However, young residents reported that they seldom visited parks in practice, which may imply that their willingness to use parks did not reflect their actual visitation behavior. Thus, our findings called attention to resurrecting outdoor activities for young residents. Because the way that young people perceive and use parks could be shaped by their values and interests [24], local policymakers should consider organizing natural programs that could cater to young residents’ orientations, which may ultimately lead to remodeling their health-related behavior [43,130].

### 5.3. Limitations and Future Research

Some potential limitations of this study are worth mentioning. First, only a single point in time was examined in this study and cause and effect could not be differentiated from a cross-sectional nature. For instance, demographic and socioeconomic characteristics may have affected the relationship between the inferred reasons and outcomes [24]. Therefore, we should use longitudinal studies that involve taking multiple measures over an extended period should be used in further research. Second, this study only depended on perceived measures instead of objective measures. The perception of green space components for health promotion was entirely based on the participants’ subjective assessment. Thus, the answers may have differed from actual results because of mental deviation from subjective perception. A previous study applied objective measures to assess associations between experiences with natural environments and mental health [131]. Similarly, a set of measures including, but not restricted to testing the biologically active components and the physical environments were used to deal with the biases. In this way, what factors that have specific health effects on young people could be further explored. Third, samples in the study mainly came from urban areas with relatively small populations. Most of the sample population were young residents with Internet access, while other young people with Internet difficulties were not included. Although the study adopted a convenient online questionnaire, it could also limit the generalizability and create a bias for the real results. Consequently, a combination of various forms of investigation such as interviews, field surveys and observations, should be applied in the future. Finally, the reasons for the differences between willingness to use parks and actual park use behavior among young residents in this study are still unclear. Although young people may be busy and prefer more attractive activities, in the future, it will be necessary to investigate the mechanism behind these differences and what features would encourage young residents to visit parks.

## 6. Conclusions

Owing to the growing disconnection between young residents and nature, in this study targeted young urban residents in China were targeted as respondents. The impact of the perception of green space for health promotion on park use willingness and behavior among young urban residents was explored. Overall, our findings demonstrated that young residents’ perception of green space components for health promotion had a greater effect on their willingness to use parks to promote health and the perception of access to green space access was the most robust predictor of willingness. In contrast, the effect of young people’s perception of green space components for health promotion was less influential on their actual park use behavior. Such disparities between park use willingness and actual use need more effort to facilitate actual park use behavior for health promotion among young residents. The findings also provided some implications for public health policymakers, urban planners and landscape architects in terms of the planning and design of green spaces oriented toward young people.

## Figures and Tables

**Table 1:** Reliability and validity analysis.

Variable	Items	Loading Value	Cronbach’s α	CR	AVE
**Green space attributes**	Access	0.801	**0.829**	**0.829**	**0.618**
	Type	0.773			
	Size	0.785			
**Landscape characteristics**	Plants	0.826	**0.902**	**0.904**	**0.703**
	Water	0.826			
	Sensory features	0.872			
	Microclimate Environments	0.828			
**Facilities**	Rest facilities	0.677	**0.756**	**0.781**	**0.645**
	Amenity facilities	0.912			

**Table 2:** Demographic characteristics of participants (N = 1135).

Characteristics	Number	Percentage (%)
Age		
18–25	687	60.5
26–30	231	20.4
31–35	217	19.1
Gender		
Male	364	32.1
Female	771	67.9
Education level		
High school or below	51	4.5
Bachelor’s degree	753	66.3
Master’s degree	282	24.8
Doctorate	49	4.3
Income		
<1000	381	33.6
1000–2000	117	10.3
2000–3000	86	7.6
3000–5000	168	14.8
5000–10,000	224	19.7
10,000–20,000	118	10.4
>20,000	41	3.6
City		
Beijing	390	34.4
Xi’an	327	28.8
Guangzhou	418	36.8
Residence location		
Central city	701	61.8
Suburban districts	390	34.4
Others	44	3.9

**Table 3:** Descriptive statistics for the dependent and independent variables (N = 1149).

Item	Mean	Standard Deviation
Dependent variables: Park use willingness and behavior		
Willingness to use parks to promote health		
Physical health	4.04	0.97
Mental health	4.42	0.87
Social health	4.00	0.99
Park use behavior		
Frequency of park visits	3.42	1.37
Duration of park visits	2.23	0.82
Independent variables: Perception of green space components for health promotion		
Green space attributes		
Access	4.26	0.90
Type	4.13	0.98
Size	4.11	0.96
Landscape characteristics		
Plants	4.19	0.93
Water	4.15	0.95
Sensory features	4.28	0.83
Microclimate environments	4.26	0.89
Facilities		
Rest facilities	3.84	1.07
Amenity facilities	4.07	0.90

**Table 4:** Correlation analyses among the variables.

	1	2	3	4	5	6	7	8	9	10	11	12	13	14	15	16	17	18	19	20
1. AGE	–																			
2. GEN	−0.196 **	–																		
3. EDU	0.147 **	−0.078 **	–																	
4. INC	0.661 **	−0.251 **	0.078 **	–																
5. CIT	0.124 **	−0.022	−0.253 **	0.087 **	–															
6. RES	−0.094 **	0.020	0.000	−0.093 **	−0.036	–														
7. ACC	0.109 **	0.043	0.081 **	0.073 *	0.006	−0.063 *	–													
8. TYP	0.080 **	0.007	0.015	0.081 **	0.116 **	−0.053	0.540 **	–												
9. SIZ	0.153 **	0.009	0.087 **	0.134 **	0.060 *	−0.019	0.586 **	0.559 **	–											
10. PLA	0.065 *	0.077 **	0.128 **	0.040	−0.004	−0.012	0.480 **	0.494 **	0.501 **	–										
11. WAT	0.012	0.030	0.073 *	0.004	0.030	−0.025	0.470 **	0.499 **	0.462 **	0.675 **	–									
12. SEN	0.035	0.057	0.054	0.030	−0.015	−0.028	0.493 **	0.447 **	0.440 **	0.619 **	0.623 **	–								
13. MIC	0.085 **	0.034	0.072 *	0.100 **	0.012	−0.029	0.483 **	0.463 **	0.470 **	0.582 **	0.564 **	0.676 **	–							
14. RES	0.097 **	0.035	0.015	0.061 *	0.049	−0.034	0.327 **	0.391 **	0.381 **	0.422 **	0.462 **	0.441 **	0.449 **	–						
15. AME	0.009	0.071 *	−0.047	0.006	0.052	−0.049	0.454 **	0.507 **	0.403 **	0.527 **	0.580 **	0.579 **	0.560 **	0.602 **	–					
16. PHY	0.084 **	−0.019	0.028	0.051	0.027	−0.035	0.538 **	0.465 **	0.513 **	0.405 **	0.410 **	0.438 **	0.433 **	0.352 **	0.434 **	–				
17. MEN	0.083 **	0.024	0.106 **	0.043	−0.032	−0.009	0.531 **	0.454 **	0.508 **	0.484 **	0.440 **	0.486 **	0.465 **	0.307 **	0.371 **	0.567 **	–			
18. SOC	0.107 **	−0.042	0.060 *	0.090 **	0.023	−0.010	0.489 **	0.490 **	0.475 **	0.428 **	0.441 **	0.385 **	0.406 **	0.354 **	0.398 **	0.576 **	0.544 **	–		
19. FRE	0.052	−0.050	−0.005	0.013	0.047	−0.027	0.152 **	0.114 **	0.130 **	0.139 **	0.109 **	0.138 **	0.142 **	0.123 **	0.161 **	0.198 **	0.137 **	0.145 **	–	
20. DUR	0.090 **	−0.027	0.017	0.099 **	−0.007	−0.024	0.045	0.069 *	0.068 *	0.113 **	0.077 **	0.115 **	0.060 *	0.081 **	0.086 **	0.039	0.092 **	0.071 *	0.062 *	–

Note: AGE—age; GEN—gender; EDU—education level; INC—income; CIT—city; RES—residence location; ACC—access; TYP—Type; SIZ—size; PLA—plants; WAT—water; SEN—sensory features; MIC—microclimate environments; RES—rest facilities; AME—amenity facilities; PHY—physical health; MEN—mental health; SOC—social health; FRE—frequency of park visits; DUR—duration of park visits. * *p* < 0.05; ** *p* < 0.01.

**Table 5:** Multiple regression results with willingness to use parks to promote health as the dependent variable.

Variable		Model 1 (Physical Health)		Model 2 (Mental Health)		Model 3 (Social Health)	
		Coefficients	VIF	Coefficients	VIF	Coefficients	VIF
**Demographic variables**	Age	0.033	1.096	0.028	1.096	0.039	1.096
	Gender	−0.058 *	1.078	−0.012	1.078	−0.064 *	1.078
	Education level	−0.035	1.794	0.029	1.794	−0.008	1.794
	Income	−0.053	1.815	−0.061 *	1.815	−0.013	1.815
**Green space attributes**	Access	0.289 **	2.215	0.301 **	2.215	0.229 **	2.215
	Type	0.109 **	2.081	0.098 **	2.081	0.151 **	2.081
	Size	0.179 **	2.158	0.172 **	2.158	0.151 **	2.158
**Landscape characteristics**	Plants	0.011	2.8 55	0.130 **	2.855	0.060	2.855
	Water	−0.017	2.859	0.029	2.859	0.174 **	2.859
	Sensory features	0.129 **	3.387	0.136 **	3.387	−0.053	3.387
	Microclimate environments	0.019	2.851	0.107 **	2.851	0.043	2.851
**Facilities**	Rest facilities	0.039	1.706	−0.020	1.706	0.038	1.706
	Amenity facilities	0.079 *	2.830	−0.045	2.830	0.012	2.830
R^2^		0.464		0.578		0.426	
Adjusted R^2^		0.458		0.573		0.419	
F-statistic		74.596 **		118.272 **		64.024 **	

Note: Independent variable—perception of green space components for health promotion. VIF—variance inflation factor. * *p* < 0.05; ** *p* < 0.01.

**Table 6:** Multiple regression results with park use behavior as the dependent variable.

Variable		Model 4 (Frequency of Park Visits)		Model 5 (Duration of Park Visits)	
		Coefficients	VIF	Coefficients	VIF
**Demographic variables**	Age	0.099 *	1.096	−0.053	1.093
	Gender	−0.067 *	1.078	0.039	1.077
	Education level	−0.042	1.794	0.045	1.788
	Income	−0.058	1.815	−0.033	1.814
**Green space attributes**	Access	0.097 *	2.215		
	Type	−0.060	2.081	−0.018	1.995
	Size	0.035	2.158	0.026	1.953
**Landscape characteristics**	Plants	0.043	2.855	−0.102 *	2.855
	Water	−0.044	2.859	0.064	2.857
	Sensory features	−0.002	3.387	−0.077	3.288
	Microclimate environments	0.052	2.851	0.120 *	2.845
**Facilities**	Rest facilities	0.015	1.706	−0.035	1.699
	Amenity facilities	0.075	2.830	0.030	2.790
R^2^		0.040		0.025	
Adjusted R^2^		0.029		0.015	
F-statistic		3.599 **		2.428 **	

Note: Independent variable—perception of green space components for health promotion. VIF—variance inflation factor. * *p* < 0.05; ** *p* < 0.01.
